# Molecular Characterization and Stress Tolerance Evaluation of New Allotetraploid Somatic Hybrids Between Carrizo Citrange and *Citrus macrophylla* W. rootstocks

**DOI:** 10.3389/fpls.2018.00901

**Published:** 2018-08-03

**Authors:** Marta Ruiz, Giovanni Pensabene-Bellavia, Ana Quiñones, Andrés García-Lor, Raphaël Morillon, Patrick Ollitrault, Eduardo Primo-Millo, Luis Navarro, Pablo Aleza

**Affiliations:** ^1^Centro de Citricultura y Producción Vegetal, Instituto Valenciano de Investigaciones Agrarias, Valencia, Spain; ^2^UMR AGAP, Centre de Coopération Internationale en Recherche Agronomique Pour le Développement, Montpellier, France

**Keywords:** rootstock breeding, protoplast fusion, genome instability, tetraploid, polyploid

## Abstract

Polyploidy is one of the main forces that drives the evolution of plants and provides great advantages for breeding. Somatic hybridization by protoplast fusion is used in citrus breeding programs. This method allows combining the whole parental genomes in a single genotype, adding complementary dominant characters, regardless of parental heterozygosity. It also contributes to surpass limitations imposed by reproductive biology and quickly generates progenies that combine the required traits. Two allotetraploid somatic hybrids recovered from the citrus rootstocks—*Citrus macrophylla* (CM) and Carrizo citrange (CC)—were characterized for morphology, genome composition using molecular markers (SNP, SSR, and InDel), and their tolerance to iron chlorosis, salinity, and *Citrus tristeza virus* (CTV). Both hybrids combine the whole parental genomes even though the loss of parental alleles was detected in most linkage groups. Mitochondrial genome was inherited from CM in both the hybrids, whereas recombination was observed for chloroplastic genome. Thus, somatic hybrids differ from each other in their genome composition, indicating that losses and rearrangements occurred during the fusion process. Both inherited the tolerance to stem pitting caused by CTV from CC, are tolerant to iron chlorosis such as CM, and have a higher tolerance to salinity than the sensitive CC. These hybrids have potential as improved rootstocks to grow citrus in areas with calcareous and saline soils where CTV is present, such as the Mediterranean region. The provided knowledge on the effects of somatic hybridization on the genome composition, anatomy, and physiology of citrus rootstocks will be key for breeding programs that aim to address current and future needs of the citrus industry.

## Introduction

The Mediterranean basin is ranked first among regions in the export of fresh market citrus fruits (FAO, 2016). This region has some adverse biotic and abiotic conditions that affect citrus cultivation. The rootstock is a key element for citrus production because it can confer tolerance to these constraints. The graft-transmissible disease tristeza, caused by the *Citrus tristeza virus* (CTV), is one of the most important limiting factors and has a strong economic impact that necessitates dramatic changes in citrus production (Cambra et al., 2000; Moreno et al., 2008). The damage brought by CTV is caused by the scion-rootstock combination, the CTV strain, and the environmental conditions (Ballester-Olmos et al., 1993). Most agricultural lands in the Mediterranean basin have two soil limiting conditions: alkalinity and, to a lesser extent, salinity. Soil alkalinity was traditionally managed using sour orange (SO) or *Citrus aurantium* L. as rootstock. Nevertheless, SO is very sensitive to Quick Decline disease caused by CTV. This limiting condition has forced the use of alternative rootstocks despite the highly desirable agronomic traits that SO induces to citrus trees. Among the main rootstocks, Cleopatra mandarin (*C. reshni* Hort. ex Tan.) and *C. macrophylla* W. (CM) are tolerant to calcareous soils, although CM is sensitive to severe CTV strains (Cambra et al., 2000). One of the main rootstocks used worldwide is Carrizo citrange (CC) [*C. sinensis* (L.) Osb. × *Poncirus trifoliata* (L.) Raf.] which is tolerant to CTV but sensitive to iron chlorosis in alkaline soils (Castle et al., 2009). Citrus is among the most salt-sensitive perennial crops (Maas, 1993). The tolerance of citrus trees to soil salinity depends greatly on the rootstock ability to restrict ion transport to the scion and this is a heritable trait (Walker, 1986). Cleopatra mandarin and CM are suited for saline soils because they restrict ion transport to the aerial part, whereas CC is sensitive to this condition as it quickly accumulates the ions and reaches toxic concentrations (Gomez-Cadenas et al., 2003). CC is considered a good rootstock for inducing high yield, big fruit size and high fruit quality to the grafted variety. CM induces vigor to citrus trees, early bearing, very high yield, and has an excellent adaptation to calcareous and saline soils. However, this rootstock is sensitive to cold temperatures, moderately sensitive to CTV, and reduces fruit quality. Therefore, CC and CM, used as citrus rootstocks, have complementary characteristics.

Rootstock breeding programs are carried out by sexual or somatic hybridization. The recovery of rootstock hybrids by sexual hybridization is hampered by citrus reproductive biology (apomixis) and the high heterozygosity of the citrus genomes. Most citrus genotypes are apomictic, except for citrons (*C. medica* L.), pummelos (*C. maxima* (L.) Osb.), clementines (*C. clementina* Hort. ex Tan.), and some mandarin hybrids. The seeds of non-apomictic genotypes generally contain only one sexual embryo, whereas the seeds of apomictic genotypes generally contain one sexual embryo and one or more nucellar embryos. The development of nucellar embryos in citrus apomictic genotypes can be initiated before fertilization, and the competition between the zygotic and nucellar embryos often results in the failure of the zygotic embryos (Wakana and Uemoto, 1987; Koltunow, 1993). In addition, the high heterozygosity level of citrus species (Herrero et al., 1996; Ollitrault et al., 2003; Barkley et al., 2006) produces a wide segregation pattern of parental traits in the progenies. The probability of having individuals that combine all the desired traits is usually very low. Therefore, a large number of individuals in these progenies need to be evaluated to find and select those that combine the desirable characteristics of the two parents. In contrast, somatic hybridization by protoplast fusion allows combining the genomes of both parents in only one genotype regardless of their level of heterozygosity, adding their dominant complementary characters (Ollitrault et al., 2000) and to overcome the sexual incompatibility between parents. This methodology is used worldwide for rootstock breeding (Grosser et al., 2000; Ollitrault et al., 2007; Dambier et al., 2011; Grosser and Gmitter, 2011).

Somatic hybridization in citrus is performed by the fusion of protoplasts derived from leaf mesophyll with protoplasts derived from embryogenic callus. In citrus, it has not yet been possible to regenerate plants from leaf protoplasts. Protoplasts isolated from embryogenic callus or leaf protoplasts that incorporate the mitochondrial genome from callus protoplasts are the only ones that have the ability to produce embryos and subsequently, plants (Kobayashi et al., 1991; Grosser and Gmitter, 2005; Guo et al., 2007). Therefore, it is necessary to have different callus lines of rootstock genotypes with favorable traits for the establishment of rootstock breeding programs based on somatic hybridization. Embryogenic callus can be easily obtained in apomictic mandarins and sweet oranges by *in vitro* ovule culture (Rangan et al., 1969; Ollitrault et al., 1994; Perez et al., 1998). However, it can be very difficult or has never been achieved in other genotypes that are essential for rootstock breeding such as SO, *P. trifoliata*, and the interspecific hybrids citranges and citrumelos (*C. paradisi* Macf. × *P. trifoliata*). Selective agents are not needed to select citrus somatic hybrids after somatic hybridizations. Instead, potential hybrids are identified among all the regenerated plants by ploidy and genetic composition analyses. The genetic analysis is often performed using a small number of molecular markers (Guo et al., 2007; Dambier et al., 2011; Grosser and Gmitter, 2011) that display the complementary allelic configuration of the parents disregarding their homogeneous distribution in the different linkage groups (LGs), hence impeding a detailed study of chromosome stability. Besides molecular analysis, a large number of plants is required for detailed physiological and agronomical evaluations of the somatic hybrids to determine their potential utility as new rootstocks (Dambier et al., 2011). Somatic hybrids go through a long juvenile phase, which often takes more than 6 years, delaying the production of seeds to obtain the plants needed to carry out the experiments (Krajewski and Rabe, 1995). This citrus juvenile phase is one of the main constraints in rootstock breeding programs. However, *in vitro* micropropagation allows the generation of a large number of clonal plants in a short time, avoiding the delay that juvenility would impose, which is a great advantage for rootstock breeding programs (Bordas et al., 2015). In this study, the genetic composition of two somatic hybrids, obtained by CM and CC protoplast fusion (Pensabene-Bellavia et al., 2015), was analyzed using single sequence repeats (SSR), single nucleotide polymorphism (SNP), and insertion or deletion (InDel) markers. Both somatic hybrids were morphologically described and their behavior was evaluated under salinity, iron deficiency, and CTV inoculation. The main objective of this study was to perform an early and detailed evaluation of the somatic hybrids to determine their potential utility as rootstocks for the Mediterranean citrus industry.

## Materials and methods

### Plant material and greenhouse conditions

Diploid CC and CM and allotetraploid somatic hybrids SMC-58 and SMC-73 were used for the experiments. Diploid CC and CM seeds were collected from the Citrus Germplasm Bank of pathogen-free plants at the Instituto Valenciano de Investigaciones Agrarias (IVIA) (Navarro et al., 2002; Navarro, 2015) and somatic hybrids SMC-58 and SMC-73 were recovered by protoplast fusion isolated from CM embryogenic callus and CC leaf mesophyll leaves (Pensabene-Bellavia et al., 2015). The somatic hybrids were micropropagated by Agromillora Research S.L. using the methodology described by Bordas et al. (2015). The seeds of CC and CM were germinated in a greenhouse using a sterile substrate composed of peat, coconut fiber, and perlite (50:25:20:5), supplemented with 1.38 g kg^−1^ of calcium superphosphate, and irrigated twice weekly with the Hoagland and Arnon (1950) nutrient solution modified for citrus (5 mM Ca(NO_3_)_2_, 1.4 mM KNO_3_, 2 mM MgSO_4_, 0.6 mM H_3_PO_4_, 20 μM Fe-EDDHA, 7.6 μM ZnSO_4_·7H_2_O, 0.50 μM CuSO_4_·5H_2_O, 50 μM H_3_BO_3_, 0.50 μMMoO_3_, and 54 μM MnSO_4_·H_2_O). The pH of the nutrient solution was adjusted to 6.0 with 1 M of KOH or H_2_SO_4_. After eight weeks, homogeneous seedlings, which were selected based on size uniformity, were transplanted individually to opaque plastic 0.5 L pots filled with a substrate composed of peat, coconut fiber, sand, and perlite (40:25:25:10). Seedlings and micropropagated plants of similar size were then randomized over the experimental area. A row of plants, not included in the experiment, was placed around the perimeter as a border. Plants were grown under greenhouse conditions with supplementary light (250 μmol m^−2^ s^−1^, 400–700 nm) to extend the photoperiod to 16 h. The temperature ranges were 16–18°C at night and 26–28°C during the day. Relative humidity (RH) was maintained at around 80%.

### Genetic characterization

Nuclear genomes were characterized using 23 SSR and 59 SNP markers selected from the 9 LG of the Clementine genetic map (Ollitrault et al., 2012a; Tables 1–4). Cytoplasmic genomes were characterized with 3 mitochondrial InDel markers, 5/*rrn*18-1 (Duminil et al., 2002), *nad*2/4-3, and *nad*7/1-2 (Froelicher et al., 2011), and 5 chloroplastic SSR markers: NTCP7, NTCP9 CCMP2, CCMP5 (Cheng et al., 2005), and CCMP6 (Bryan et al., 1999; Weising and Gardner, 1999; Table 4). All the analyses were performed in the somatic hybrids, the parents (CM and CC), and the CM embryogenic callus used for protoplast fusion. For these characterizations, genomic DNA was isolated using the methodology described by Dellaporta et al. (1983) with few modifications (0.5 M EDTA, pH 8.0, 1 M Tris-HCl, pH 8.0, 5 M NaCl, 2% MATAB, 1% PEG 6000 and 0.5% Na_2_SO_3_) and was performed using different DNA extractions from leaves of different branches of both somatic hybrids.

**Table 1:** Molecular markers analyzed indicating the type of marker, locus name, linkage groups (LGs) 1 and 2 and location within LG in centimorgans (cM), bibliographic reference in the literature, and GeneBank accession.

**Type**	**Locus**	**Location**		**References**	
		**LG**	**cM**	**Bibliography**	**GeneBank**
SNP	^*^CiC4827-01	1	20.5	Ollitrault et al., 2012b	ET072918
SNP	CiC2110-01	1	28.8	Ollitrault et al., 2012b	ET099643
SSR	CiBE5720	1	58.5	Ollitrault et al., 2010b	ET082224
SNP	^*^CiC4581-01	1	63.7	Ollitrault et al., 2012b	ET109034
SNP	^*^ACO-P353	1	80.4	Ollitrault et al., 2012a	JX630066
SNP	ACO-C601	1	83.4	Ollitrault et al., 2012a	JX630065
SNP	CiC0599-01	1	102.4	Ollitrault et al., 2012b	ET093125
SNP	TSC-C80	1	111.6	García-Lor et al., 2013a	JX630084
SSR	JK-taa15	1	119.7	Kijas et al., 1997	none
SNP	^*^F3H-M309	2	19.6	García-Lor et al., 2013a	JX630066
SNP	^*^F3H-C341	2	20.0	García-Lor et al., 2013a	JX630067
SNP	^*^F3H-P30	2	20.0	García-Lor et al., 2013a	JX630066
SNP	^*^PEPC-M316	2	32.6	García-Lor et al., 2013a	JX630067
SNP	PEPC-C328	2	32.6	García-Lor et al., 2013a	JX630067
SSR	mCrCIR07D05	2	75.6	Cuenca et al., 2011	FR677574
SNP	SOS1-M50	2	78.5	García-Lor et al., 2013a	JX630068
SNP	^*^CiC3712-01	2	93.9	Ollitrault et al., 2012b	ET079481
SNP	CCC1-P727	2	110.9	García-Lor et al., 2013a	JX630069
SNP	^*^CCC1-M85	2	110.9	García-Lor et al., 2013a	JX630069
SSR	JK-TAA41	2	131.8	Kijas et al., 1997	none
SNP	^*^PKF-C64	2	131.2	García-Lor et al., 2013a	JX630076
SNP	TRPA-M593	2	132.3	García-Lor et al., 2013a	JX630070
SNP	PKF-M186	2	133.5	García-Lor et al., 2013a	JX630076

* *Non-polymorphic markers*.

**Table 2:** Molecular markers analyzed indicating the type of marker, locus name, linkage groups (LGs) 3, 4, and 5 and location within LG in centimorgans (cM), bibliographic reference in the literature, and GeneBank accession.

**Type**	**Locus**	**Location**		**References**	
		**LG**	**cM**	**Literature**	**GeneBank**
SNP	INVA-M437	3	30.2	García-Lor et al., 2013a	JX630071
SNP	MDH-M519	3	34.8	García-Lor et al., 2013a	JX630072
SNP	^*^MDH-MP69	3	34.8	García-Lor et al., 2013a	JX630072
SNP	^*^CiC4681-02	3	92.8	Ollitrault et al., 2012b	ET109640
SNP	NCED3-M535	3	101.3	García-Lor et al., 2013a	JX630086
SNP	CiC5796-12	3	109.9	Ollitrault et al., 2012b	ET0822752
SNP	ATMR-M728	3	141.9	García-Lor et al., 2013a	JX630073
SNP	ATMR-C372	3	141.9	García-Lor et al., 2013a	JX630073
SSR	Ci08A10	3	144.9	Froelicher et al., 2011	AJ567414
SNP	CHS-M183	3	167.3	García-Lor et al., 2013a	JX630074
SNP	^*^CHS-P57	3	167.3	García-Lor et al., 2013a	JX630074
SNP	^*^CiC4240-04	4	7.1	Ollitrault et al., 2012b	ET106812
SNP	CHI-M598	4	11.0	García-Lor et al., 2013a	JX630075
SSR	mCrCIR07D06	4	16.3	Cuenca et al., 2011	FR677581
SNP	CiC2840-01	4	17.0	Ollitrault et al., 2012b	ET103429
SNP	CiC3740-02	4	43.9	Ollitrault et al., 2012b	ET079647
SSR	mCrCIR03G05	4	75.1	Cuenca et al., 2011	FR677578.1
SNP	^*^CiC6213-07	4	85.5	Ollitrault et al., 2012b	ET085253
SNP	CiC1380-05	5	17.2	Ollitrault et al., 2012b	ET072553
SNP	CiC5788-16	5	41.5	Ollitrault et al., 2012b	ET082679
SNP	^*^CiC5842-02	5	77.3	Ollitrault et al., 2012b	ET083106
SNP	^*^NADK2-M285	5	86.0	García-Lor et al., 2013a	JX630077
SSR	mCrCIR06A12	5	98.7	Froelicher et al., 2011	AM489742
SNP	DFR-M240	5	105.7	García-Lor et al., 2013a	JX630074

* *Non-polymorphic markers*.

**Table 3:** Molecular markers analyzed indicating the type of marker, locus name, linkage groups (LGs) 6 and 7 and location within LG in centimorgans (cM), bibliographic reference in the literature, and GeneBank accession.

**Type**	**Locus**	**Location**		**References**	
		**LG**	**cM**	**Literature**	**GeneBank**
SSR	^*^mCrCIR04H09	6	0.0	Ollitrault et al., 2012a	FR692370
SNP	CiC4356-06	6	6.2	Ollitrault et al., 2012b	ET107540
SSR	MEST132	6	26.9	Aleza et al., 2011	DY276930
SSR	CiBE4818	6	28.3	Ollitrault et al., 2010b	ET110604
SSR	CiBE0733	6	42.2	Ollitrault et al., 2010b	ET094202
SNP	^*^CiC2128-01	6	61.2	Ollitrault et al., 2012b	ET111354
SSR	mCrCIR02B11	6	69.2	Ollitrault et al., 2012a	FR692358
SNP	PSY-M30	6	69.7	García-Lor et al., 2013a	JX630080
SNP	PSY-C461	6	69.7	García-Lor et al., 2013a	JX630080
SNP	CiC3056-02	6	70.5	Ollitrault et al., 2012b	ET075329
SSR	^*^CiBE6256	6	84.6	Ollitrault et al., 2010b	ET085615
SNP	^*^AocM290	6	85.9	Ollitrault et al., 2012b	JX630079
SNP	AocC593	6	85.9	Ollitrault et al., 2012b	DY293375
SSR	MEST123	6	93.0	Aleza et al., 2011	DY276100
SSR	CiBE5866	6	99.8	Ollitrault et al., 2010b	ET083232
SSR	mCrCIR07E05	7	13.1	Froelicher et al., 2011	AM489749
SNP	^*^CiC1444-03	7	13.6	Ollitrault et al., 2012b	ET073216
SNP	DXS-M618	7	40.7	García-Lor et al., 2013a	JX630082
SNP	DXS-C545	7	40.7	García-Lor et al., 2013a	JX630082
SNP	FLS-P129	7	46.0	García-Lor et al., 2013a	JX630083
SSR	^*^mCrCIR03E06	7	75.1	Ollitrault et al., 2012a	FR692363
SSR	Ci07C07	7	98.0	Froelicher et al., 2011	AJ567409

* *Non-polymorphic markers*.

**Table 4:** Molecular markers analyzed indicating the type of marker, locus name, linkage groups (LGs) 8 and 9 and location within LG in centimorgans (cM), bibliographic reference in the literature, and GeneBank accession.

**Type**	**Locus**	**Location**		**References**	
		**LG**	**cM**	**Literature**	**GeneBank**
SSR	mCrCIR07B05	8	31.7	Froelicher et al., 2011	AM489747
SSR	CiBE0214	8	40.4	Ollitrault et al., 2010b	ET088913
SNP	CiC5164-02	8	45.6	Ollitrault et al., 2012b	ET111943
SNP	CiC1749-05	8	103	Ollitrault et al., 2012b	ET097636
SNP	^*^CiC4876-07	9	2.7	Ollitrault et al., 2012b	ET080580
SNP	^*^CiC5087-01	9	15.9	Ollitrault et al., 2012b	ET111514
SSR	mCrCIR07F11	9	49.6	Kamiri et al., 2011	FR677567
SNP	CiC2518-02	9	53.5	Ollitrault et al., 2012b	ET101955
SSR	Ci08C05	9	55.1	Froelicher et al., 2011	AJ567415
SNP	^*^LCYB-P736	9	78.9	García-Lor et al., 2013a	JX630084
SNP	LCYB-M480	9	78.9	García-Lor et al., 2013a	JX630084
SNP	HYB-M62	9	102.3	García-Lor et al., 2013a	AF315289
SNP	HYB-C433	9	102.3	García-Lor et al., 2013a	JX630087
Indel	*nad*2/4-3	Mitocondrial		Froelicher et al., 2011	
Indel	^*^ nad7/1-2	Mitocondrial		Froelicher et al., 2011	
Indel	^*^ 5/*rrn*18-1	Mitocondrial		Duminil et al., 2002	
cpSSR	CCMP2	Chloroplastic		Cheng et al., 2003	
cpSSR	CCMP5	Chloroplastic		Cheng et al., 2003	
cpSSR	CCMP6	Chloroplastic		Cheng et al., 2003	
cpSSR	NTCP7	Chloroplastic		Cheng et al., 2003	
cpSSR	^*^ NTCP9	Chloroplastic		Cheng et al., 2003	

* *Non-polymorphic markers*.

### SSR markers

PCR amplifications were performed using Thermocycler ep gradient S (Eppendorf®, Germany) in 10 μL final volume, containing 0.8 U of *Taq* DNA polymerase (Fermentas®, Germany), 10 ng of citrus template DNA, 0.2 mM wellRED (Sigma®, Germany) dye-labeled forward primer, 0.2 mM non-dye-labeled reverse primer, 0.2 mM each dNTP, and PCR reaction buffer 10X composed of 750 mM Tris-HCl, pH 9.0, 50 mM KCl, 200 mM (NH_4_)_2_SO_4_, 1.5 mM MgCl_2_, and 0.0001% BSA. The cycling program was set as follows: denaturation for 5 min at 94°C followed by 40 repeats of 30 s at 94°C, 1 min at the annealing temperature of each primer pair, 45 s at 72°C, and a final elongation step of 4 min at 72°C. Capillary electrophoresis was carried out using a CEQ™ 8,000 Genetic Analysis System (Beckman Coulter Inc., USA). The PCR products were initially denatured for 2 min at 90°C, injected for 30 s at 2 kV, and subsequently separated for 35 min at 6 kV. Alleles were sized based on a DNA size standard (400 bp). The GenomeLab™ GeXP v.10.0 genetic analysis software was used for data collection. Allele dosage was calculated using MAC-PR (microsatellite DNA allele counting-peak ratio) method (Esselink et al., 2004), validated in citrus by Cuenca et al. (2011).

### SNP markers

Genetic analysis of SNP markers was performed using KASPar technology by LGCgenomics (http://www.lgcgenomics.com). Primers were designed by LGCgenomic from each SNP locus flanking sequence (approximately 50 nt on each side of the SNP). The KASPar genotyping system is a competitive allele-specific dual Förster Resonance Energy Transfer (FRET)-based assay for SNP genotyping. A detailed description of specific conditions and reagents can be found in Cuppen (2007). Identification of allele dosage in heterozygous somatic hybrids was carried out based on the relative allele signals described by Cuenca et al. (2013) and Aleza et al. (2015).

### Identification of the genetic structure of somatic hybrids and their parents

Allelic configurations of the somatic hybrids SMC-58 and SMC-73 and their parents, CM and CC, were determined using the SSR and SNP genotyping data. For markers showing different alleles for the parents (A_1_A_2_ + A_3_A_4_, and A_1_A_2_ + A_3_A_3_), the somatic hybrids genotype was directly annotated. In the case of parents sharing alleles for a given marker (A_1_A_2_ + A_2_A_2_ o A_1_A_2_ + A_2_A_3_), the allelic configuration of the somatic hybrids was based on the estimated allele dosage.

### Morphologic characterization

Plant morphology was evaluated on 9-month-old plants that were cultivated in the greenhouse under the above described conditions. Twelve plants of CC, CM, and the somatic hybrids (SMC-58 and SMC-73) were chosen for performing the evaluation. Measurements were taken on plant height, internodal length, and leaf number. Leaf greenness of 3 mature leaves was measured in each plant using a SPAD device (Minolta®, Japan) and the mean value of 5 readings was taken. The length (l) and width (w) of the main leaflet were also registered in the same leaves. Leaf index, representative of leaf shape, was calculated from l/w relations. Additionally, all the relevant International Plant Genetic Resources Institute (IPGRI) descriptors for each genotype were annotated (IPGRI, 1999).

### Iron chlorosis tolerance evaluation

Twelve homogeneous plants of each genotype were trimmed to a single stem and transplanted to 0.5 L pots and then grouped into two groups based on substrate type. Control substrate was composed of peat and sand (2:3) with added 0.4% (w/v) Ca(H_2_PO_4_)_2_, whereas chlorosis-inducing substrate had additional 10% (v/v) of CaCO_3_ added to the mix. Plants were previously acclimated and were maintained for 4 weeks under the irrigation and climatic conditions previously described. Any new lateral branching shoots were detected and eliminated every 3 days to focus the growth in a single shoot. A plastic ring was placed on top of the stem to differentiate the newly developed biomass prior to the initiation of irrigation treatments. Plants growing on normal substrate were irrigated with the solution previously described, which contained 20 μM Fe-EDDHA, and were chosen as the control treatment (Ct). Plants growing on the chlorosis-inducing substrate were irrigated with a similar solution than Ct treatment but deficient in iron (2 μM Fe-EDDHA) and containing carbonates (10 mM NaHCO_3_). These conditions were considered the chlorosis-inducing treatment (Ch). Plants were randomized over the experimental area with a guard row and irrigated twice weekly for 10 weeks. After treatments, the new shoot was taken from each plant, rinsed with deionized water, and separated into leaves and stems. They were then fresh-weighed individually and dried in a forced draft oven at 70°C for 48 h until constant dry weight (DW) was obtained. Plant growth was measured using the shoot (leaf and stem) DW and iron content analysis was performed using leaves. The chlorophyll content in leaves was monitored by measuring changes in leaf greenness with a SPAD chlorophyll meter (Minolta, Japan). Two fully expanded leaves per plant were marked with labels and five readings were taken per leaf, avoiding the midrib, at the initial and final days of the trial period. Leaf greenness index was calculated as the ratio of final/initial SPAD readings. Values below 1 indicate greenness descent over the trial period (Castle et al., 2009). The iron concentration was measured from dry tissues (0.5 g) that were burnt in a muffle furnace for 12 h at 550°C. Iron was extracted with 2% nitric acid (Hiperpur, Panreac) in an ultrasonic bath (Fungilab®, Spain) for 30 min at 40°C and the concentration was measured using atomic absorption spectrometry in an ASS Analyst200 (Perkin Elmer®, USA).

### Salinity tolerance evaluation

Forty homogeneous plants of each genotype trimmed to a single stem were selected and divided into groups that were irrigated with the basal nutrient solution described above. Either 0 (control, Ct) or 40 mM NaCl (salt-treated, +S) was added to each group. Pots were irrigated with 400 mL of solution per pot every 3 days. Excess solution was drained out of the pot to avoid salt accumulation in the substrate. A plastic ring was placed at the top of the stems to differentiate the newly developed biomass before irrigation treatments were initiated. Leaf gas exchange parameters were registered weekly using a portable infra-red gas analyzer LCpro+ (ADC Bioscientific Ltd., UK). Net CO_2_ assimilation (*A*_CO2_) and transpiration (*E*) rates were monitored between 10 a.m. and 2 p.m. The measurements were taken in two mid-stem leaves of 9 plants per treatment and genotype by taking 3 consecutive measurements on each leaf. Photosynthetically active radiation (PAR) at the leaf surface was adjusted to 1,000 μmol m^−2^ s^−1^, which exceeds the saturating value for citrus, and atmospheric CO_2_ concentration was not manipulated. Relative humidity and temperature in the greenhouse were recorded during each measurement event and were maintained by the conditions previously described. Dry weight of new leaves, leaf abscission percentage, and leaf burned area percentage were evaluated on leaves after 20 days of salt treatment and 5 mid-stem leaves, roots, and stems were sampled for analysis. Plant organs were rinsed with deionized water and 10% (w/v) Tween 20 (Sigma-Aldrich Co., Germany) and dried in a forced draft oven at 65°C for 48 h until reaching constant DW. Dried samples were crushed separately in a hammer-mill and were stored at room temperature to determine iron concentration in different organs. Chloride (Cl^−^) was determined by silver ion titration using a Corning 926 chloridometer (Corning®) as described by Gilliam (1971). Sodium (Na^+^) and potassium (K^+^) concentration were determined by inductively coupled plasma atomic emission spectroscopy (ICP-AOES iCAP 6000, Thermo Scientific). Samples (0.5 g) were pre-digested overnight with 2% HNO_3_ and 0.1% (w/v) Triton-X 100 (Sigma-Aldrich Co.) prior to processing on a digestion block at 120°C. The digestion tubes were then removed and cooled at room temperature. 2.0 mL of a 70% ultra-trace-metal-grade HClO_4_ was then added to the sample and heated at 220°C until white fumes were produced. Digest was diluted to a 25 mL with ultrapure water (Campbell and Plank, 1998) and filtered in n° 1 Whatman paper.

### CTV tolerance evaluation

Six plants of CC, CM, SMC-58, and SMC-73 were inoculated by bark grafting with CTV T388 strain (+CTV), which is a very aggressive strain to CM (Moreno et al., 1990; Ballester-Olmos et al., 1993). The inoculum was obtained from the IVIA citrus virus and virus-like collection. After 25 days, plants were pruned leaving 5 cm above the inoculum to induce a new shooting. Control treatment (Ct) was applied to 3 plants that were not inoculated with the virus. The plants were cultivated under the above-described greenhouse conditions for 12 months. Plant size and the weight of roots and aerial parts were registered, and CTV symptoms were evaluated in leaves and stem wood.

### Statistical analysis

Data were subjected to analysis of variance (ANOVA). Means were separated using Duncan's multiple range test at *P* < 0.05 with the Statgraphics Plus, version 5.1 (Statistical Graphics, Englewood Cliffs) software.

## Results

### Genetic characterization

Genetic analysis of the CM callus used for protoplast fusion did not show any differences when compared to the tree of the IVIA Citrus Germplasm Bank for the 34 SSR markers analyzed (Pensabene-Bellavia, 2009). Somatic hybrids SMC-58 and SMC-73 and their parents (CC and CM) were analyzed using 90 markers (82 from the nuclear genome and 8 from the cytoplasmic genome). Fifty-nine of the markers analyzed in nuclear genome were SNPs, whereas 23 were SSRs. All of them were distributed on the 9 LGs of the reference genetic citrus map (Ollitrault et al., 2012a) with a coverage between 5 and 15 markers per LG. Thirty six of the fifty-nine SNPs analyzed were polymorphic between parents, whereas polymorphism was found in 20 of the 23 SSRs analyzed (Tables 1–4).

**Figure 1:**
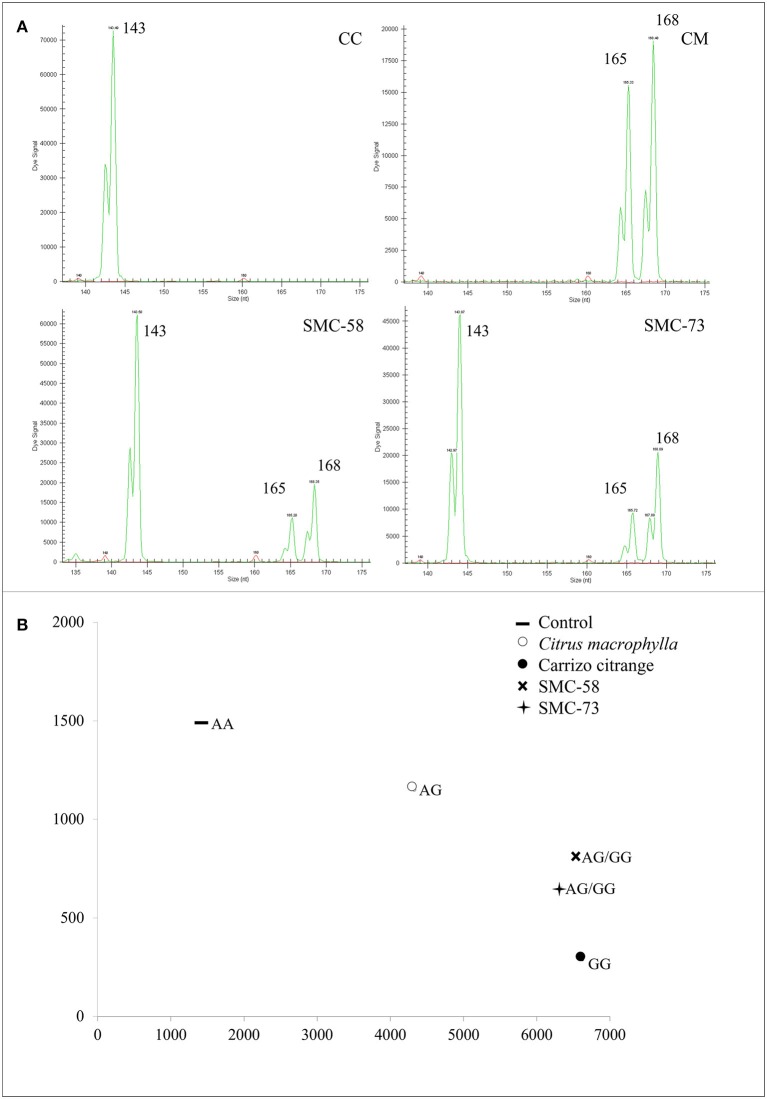
Allelic configurations on LG 1 that correspond to the addition of both parents. **(A)** Electropherograms of the somatic hybrids SMC-58 and SMC-73 displaying four different alleles from their parents *C. macrophylla* (CM) and Carrizo citrange (CC) using JK-TAA15 SSR marker. Numbers indicate the size of the amplified allele in nucleotides (nt). **(B)** Plot of allele signals of ACO-C601 SNP marker in somatic hybrids and their parents. CM displayed AG alleles, Carrizo citrange displayed GG alleles and somatic hybrids display AGGG alleles. Letters indicate the allelic configuration for each genotype as: A, adenine; C, cytosine; T, thymine; G, guanine.

**Figure 2:**
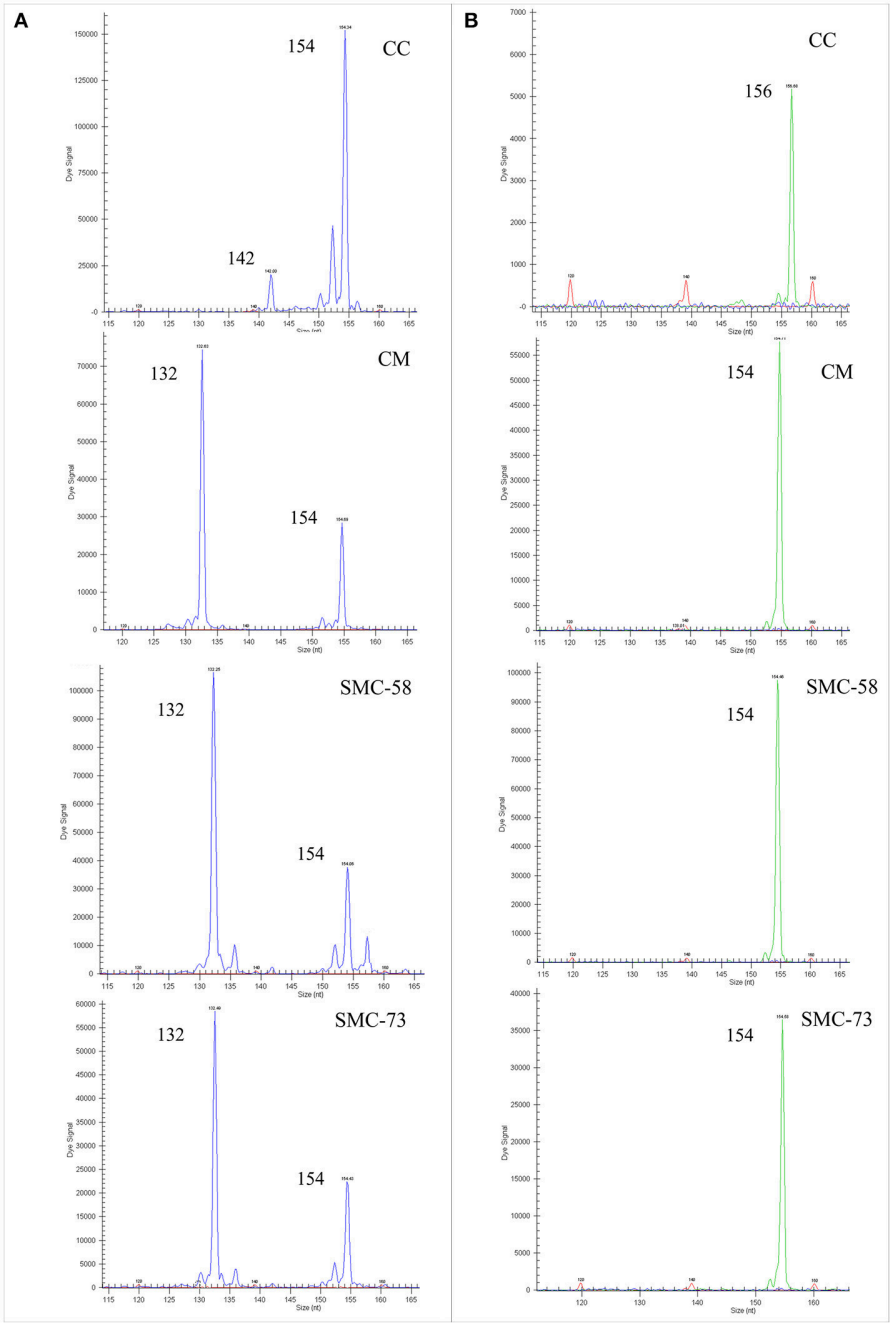
Electropherograms of the somatic hybrids SMC-58 and SMC-73 and their parents *C. macrophylla* (CM) and Carrizo citrange (CC) displaying allelic losses. **(A)** JK-TAA41 SSR marker on LG 2. **(B)** Ci08A10 SSR marker on LG 3. Numbers indicate the size of the amplified allele in nucleotides (nt).

**Figure 3:**
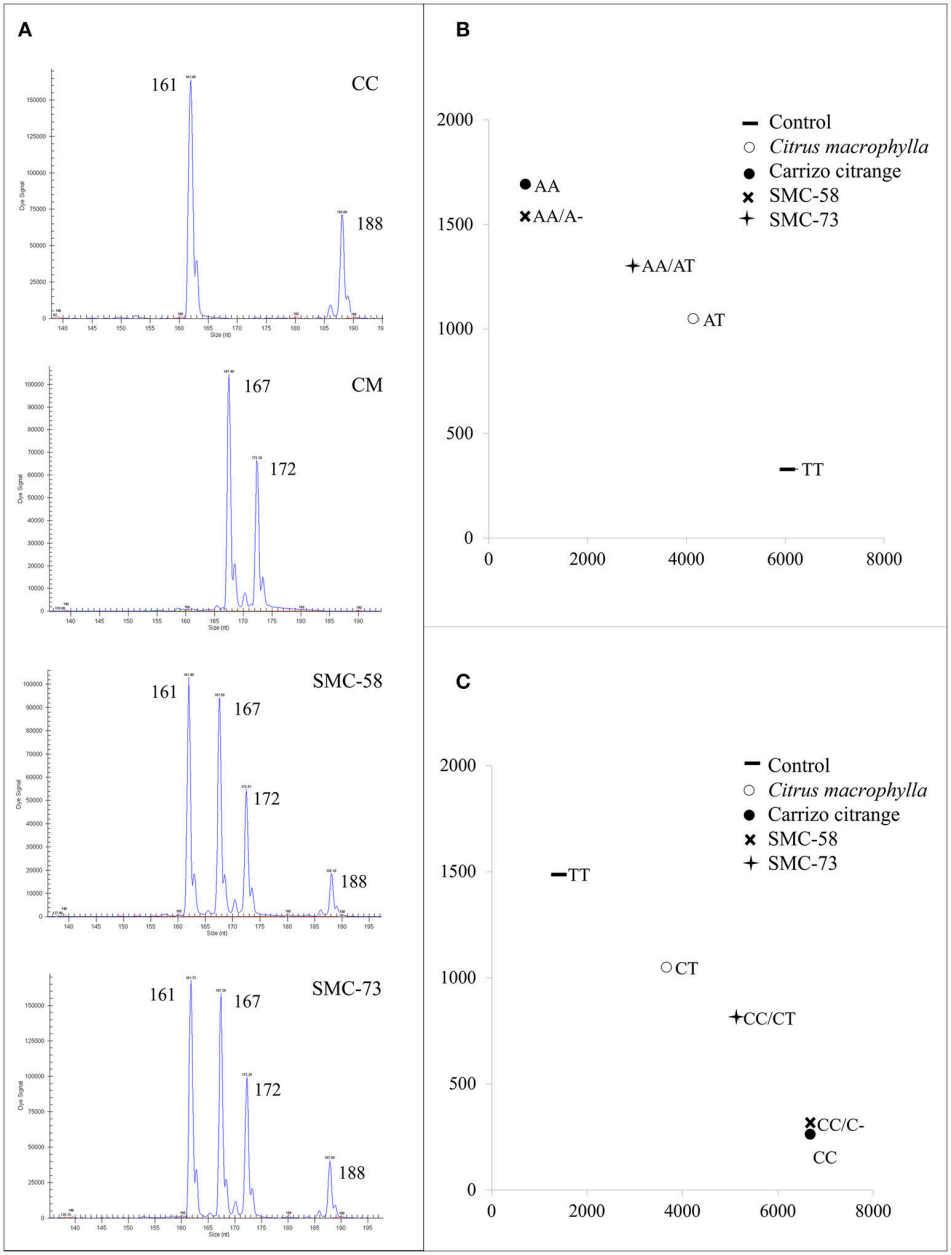
Allelic configurations of the somatic hybrids SMC-58 and SMC-73 and their parents *C. macrophylla* (CM) and Carrizo citrange (CC). **(A)** Ci07D06 SSR marker on LG 4 where somatic hybrids display four different alleles from their parents. Numbers indicate the size of the amplified allele in nucleotides (nt). Plot of allele signals of **(B)** PSY-C461 and **(C)** AocC593 SNP markers on LG 6. For PSY-C461 SNP marker, CM displayed AT alleles, Carrizo citrange displayed AA alleles, SMC-58 AAA alleles with the loss of T allele from CM and SMC-73 with the alleles of both parents, CM, and CC. For AocC593, CM displayed CT alleles, Carrizo citrange displayed CC alleles, SMC-58 CCC alleles with the loss of T allele from CM and SMC-73 with the alleles of both parents, CM and CC. Letters indicate the allelic configuration for each genotype as: A, adenine; C, cytosine; T, thymine; G, guanine.

The LG 1 was analyzed with 7 polymorphic markers, consisting of 5 SNPs and 2 SSRs (Table 5). Somatic hybrids SMC-58 and SMC-73 displayed allelic configurations that correspond with the addition of both genome parents as seen with the JK-TAA15 SSR marker (Figure 1A) or the ACO-C601 SNP marker (Figure 1B). The LG 2 was analyzed using 5 SNP markers and 2 SSRs that were polymorphic between parents. The addition of the alleles from both parents was observed in the two somatic hybrids for all the analyzed markers, except for the JK-TAA41 SSR marker (Figure 2A) that showed the loss of the 142 nt allele from CC in both somatic hybrids. The LG 3 was analyzed using 6 SNPs and 1 SSR marker that were polymorphic between parents. Somatic hybrids combined all the alleles from each parent, except for Ci08A10 SSR marker (Figure 2B) that lost the 156 nt allele from CC in both somatic hybrids. The LG 4 was analyzed using 3 SNPs and 2 SSR markers (Table 6) and both somatic hybrids combined all the parental alleles. Figure 3A shows results obtained for Ci07D06 SSR marker as an example. The LG 5 was analyzed using 3 SNPs and 1 SSR marker and results did not show allelic losses in these loci. The LG 6 was analyzed using 5 SNPs and 6 SSR markers. Somatic hybrids showed allelic losses in 5 of the 6 SSR markers analyzed (CiBE4818, CiBE0733, mCrCIR02B11, MEST123, and CiBE5866). The origin of lost alleles was CM, except for the locus CiBE0733 that lost the CC allele. Three of these losses were shared between the somatic hybrids, whereas 1 and 2 of them affected SMC-58 and SMC-73, respectively. Besides, on 3 of the 5 SNP markers (CiC4356-06, PSY-C461, and AocC593), the SMC-58 somatic hybrid lost the T allele from CM, whereas these differences were not observed in SMC-73 (Table 6). Figures 3B and C are examples of the results obtained for the PSY-C461 and AocC593 SNP markers, displaying the T allele lost in the SMC-58 somatic hybrid. The LG 7 was analyzed with 3 SNPs and 2 SSR markers. The 239 nt allele from CM was lost on Ci07C07 SSR locus in both somatic hybrids. On the LG 8, 2 SNPs and 2 SSR markers were analyzed, whereas on the LG 9, 4 SNPs and 2 SSR markers were used. In both LGs, the hybrids displayed allelic configurations that correspond with the addition of both genome parents (Table 7).

**Table 5:** Genetic analysis using SNP and SSR nuclear markers located on LGs 1, 2, and 3 performed on SMC-58 and SMC-73 somatic hybrids that were obtained by protoplast fusion between *C. macrophylla* (CM) and Carrizo citrange (CC).

**Locus**	**LG**	**CC**		**CM**		**SMC-58**				**SMC-73**			
CiC2110-01	1	*^*y*^*C	A	A	A	C	A	A	A	C	A	A	A
CiBE5720		*^*z*^*308	329	308	320	308	329	308	320	308	329	308	320
ACO-C601		G	G	A	G	G	G	A	G	G	G	A	G
CiC0599-01		G	A	G	G	G	A	G	G	G	A	G	G
TSC-C80		G	G	G	T	G	G	G	T	G	G	G	T
JK-TAA15		143		165	168	143		165	168	143		165	168
PEPC-C328		A	A	A	G	A	A	A	G	A	A	A	G
mCrCIR07D05	2	189		195		189		195		189		195	
SOS1-M50		A	G	A	A	A	G	A	A	A	G	A	A
CCC1-P727		C	T	C	C	C	T	C	C	C	T	C	C
JK-TAA41		142	154	132	154	142	154	132	154	142	154	132	154
TRPA-M593		C	G	C	C	C	G	C	C	C	G	C	C
PKF-M186		T	T	C	T	T	T	C	T	T	T	C	T
INVA-M437		C	T	C	C	C	T	C	C	C	T	C	C
MDH-M519	3	C	T	C	C	C	T	C	C	C	T	C	C
NCED3-M535		G	T	T	T	G	T	T	T	G	T	T	T
CiC5796-12		A	A	C	C	A	A	C	C	A	A	C	C
ATMR-M728		T	T	G	G	T	T	G	G	T	T	G	G
ATMR-C372		A	A	A	G	A	A	A	G	A	A	A	G
Ci08A10		156		154		156		154		156		154	
CHS-M183		C	C	G	G	C	C	G	G	C	C	G	G

y *SNP alleles: A, adenine; C, cytosine; T, thymine; G, guanine*.

z *SSR allele: Numbers are the allele size in nucleotides. Lost alleles are marked in gray*.

**Table 6:** Genetic analysis using SNP and SSR nuclear markers located on LGs 4, 5, and 6 performed on SMC-58 and SMC-73 somatic hybrids that were obtained by protoplast fusion between *C. macrophylla* (CM) and Carrizo citrange (CC).

**Locus**	**LG**	**CC**		**CM**		**SMC-58**				**SMC-73**			
CHI-M598	4	*^*y*^*C	C	G	C	C	C	G	C	C	C	G	C
mCrCIR07D06		*^*z*^*162	188	167	172	162	188	167	172	162	188	167	172
CiC2840-01		T	T	C	C	T	T	C	C	T	T	C	C
CiC3740-02		G	C	G	G	G	C	G	G	G	C	G	G
mCrCIR03G05		213	219	199		213	219	199		213	219	199	
CiC1380-05	5	T	T	C	C	T	T	C	C	T	T	C	C
CiC5788-16		G	A	A	A	G	A	A	A	G	A	A	A
mCrCIR06A12		92	103	86		92	103	86		92	103	86	
DFR-M240		C	G	C	C	C	G	C	C	C	G	C	C
CiC4356-06	6	C	C	C	T	C	C	C	T	C	C	C	T
MEST132		231	244	244		231	244	244		231	244	244	
CiBE4818		151	162	154		151	162	154		151	162	154	
CiBE0733		240	245	235		240	245	235		240	245	235	
mCrCIR02B11		232		232	248	232		232	248	232		232	248
PSY-M30		C	G	G	G	C	G	G	G	C	G	G	G
PSY-C461		A	A	A	T	A	A	A	T	A	A	A	T
CiC3056-02		G	A	A	A	G	A	A	A	G	A	A	A
AocC593		C	C	C	T	C	C	C	T	C	C	C	T
MEST123		239	246	250		239	246	250		239	246	250	
CiBE5866		214		222		214		222		214		222	

y *SNP alleles: A, adenine; C, cytosine; T, thymine; G, guanine*.

z *SSR allele: Numbers are the allele size in nucleotides. Lost alleles are marked in gray*.

**Table 7:** Genetic analysis using SNP and SSR nuclear markers located on LGs 7, 8, and 9 and mitochondrial (mt) and chloroplastic (cp) markers performed on SMC-58 and SMC-73 somatic hybrids that were obtained by protoplast fusion between *C. macrophylla* (CM) and Carrizo citrange (CC).

**Locus**	**LG**	**CC**		**CM**		**SMC-58**				**SMC-73**			
mCrCIR07E05	7	*^*z*^*119	128	116		119	128	116		119	128	116	
DXS-M618		*^*y*^*G	G	A	A	G	G	A	A	G	G	A	A
DXS-C545		G	G	C	G	G	G	C	G	G	G	C	G
FLS-P129		C	T	T	T	C	T	T	T	C	T	T	T
Ci07C07		212		239		212		239		212		239	
mCrCIR07B05	8	196		203	210	196		203	210	196		203	210
CiBE0214		312		309		312		309		312		309	
CiC5164-02		C	C	T	T	C	C	T	T	C	C	T	T
CiC1749-05		G	T	T	T	G	T	T	T	G	T	T	T
mCrCIR07F11	9	160		162	164	160		162	164	160		162	164
CiC2518-02		T	A	T	T	T	A	T	T	T	A	T	T
Ci08C05		153		153	156	153		153	156	153		153	156
LCYB-M480		T	C	T	T	T	C	T	T	T	C	T	T
HYB-M62		A	A	C	C	A	A	C	C	A	A	C	C
HYB-C433		G	G	A	G	G	G	A	G	G	G	A	G
*nad*2/4-3	mt	261		251		251				251			
CCMP2	cp	197		203		197		203		197			
CCMP5	cp	93		95		93		95		93			
CCMP6	cp	133		135		133		135		133			
NTCP7	cp	182		188		182		188		182			

y *SNP alleles: A, adenine; C, cytosine; T, thymine; G, guanine*.

z *SSR allele: numbers are the allele size in nucleotides. Lost SSR alleles and modified SNP alleles are marked in gray*.

In summary, somatic hybrids SMC-58 and SMC-73 combine the parental alleles from CC and CM in 45 of the 56 nuclear markers analyzed (80%). However, allelic losses were found in 11 of the loci analyzed. The origin of lost alleles was CM in 8 loci and CC in 3 loci. Most of the lost alleles, 8 of the 11, were located on the LG 6 (Table 6) and 2 of them have a CC origin, whereas 6 come from CM. The rest of lost alleles were located on LGs 2, 3 (Table 5), and 7 (Table 7). On LGs 2 and 3, the origin of lost alleles was CC, whereas on LG 7, the origin of lost alleles was CM. We found alleles that are lost only in one or the other when comparing the genetic configuration of both somatic hybrids. Therefore, they are genetically different. SMC-58 lost 3 SNP alleles from CM (Figures 3B and C) that were identified in SMC-73 and SMC-73 lost 2 SSR alleles (mCrCIR02B11 and MEST 123 loci), one from CM and the other one from CC, although these alleles were present in the SMC-58 somatic hybrid. We investigated the parental origin of these 11 lost alleles. CM is a hybrid of *C. micrantha* W. and *C. medica* (Curk et al., 2016), two of the *Citrus* ancestral species. CC has *C. sinensis* and *P. trifoliata* in its pedigree and *C. sinensis* is a secondary species that originated from crosses between *C. maxima* and *C. reticulata* (García-Lor et al., 2013b). In Ci08A10, Cibe4818, and Cibe5866 SSR markers, we cannot decipher the parental origin of the lost alleles because Ci08A10 is homozygous for CC and for the last 2 markers, the lost allele is shared between *C. micrantha* and citron. Regarding Ci07C07 SSR marker and CiC4356-06, PSY-C461 and AoC-C593 SNP markers, the lost allele comes from citron, whereas for mCrCIR02B11, JK-TAA41, and Cibe0733 SSR markers lost alleles come from *C. micrantha, P. trifoliata*, and *C. sinensis*, respectively. The later lost allele is shared between *C. maxima* and *C. reticulata* parental species of sweet orange.

**Figure 4:**
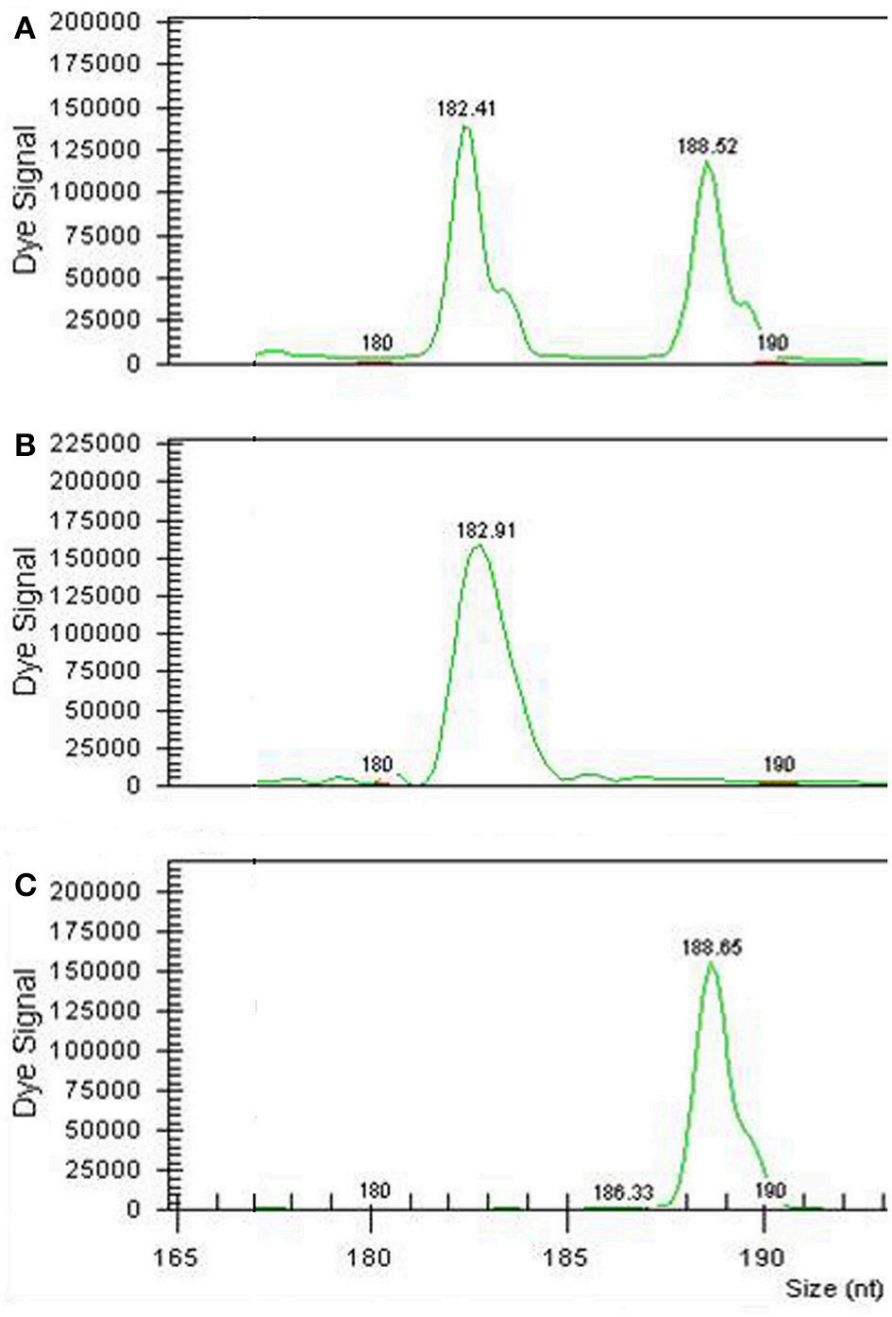
Electropherogram of the chloroplastic SSR marker NTCP7 analyzed in the SMC-58 somatic hybrid **(A)** and his parents CC **(B)** and CM **(C)** Numbers mean the size in nucleotides of each amplified allele.

The cytoplasmic genome was analyzed using 1 mitochondrial marker (*nad*2/4-3) and 4 chloroplastic markers (CCMP*2*, CCMP*5, CCMP6*, and NTCP*7*) (Table 8) that were polymorphic between CC and CM. Both somatic hybrids had the 251 nt allele of the mitochondrial marker *nad*2/4-3 that belongs to the embryogenic parental CM. However, chloroplastic genome analysis showed that the origin of SMC-73 chloroplasts was CC for the 4 markers analyzed, whereas SMC-58 combined both CM and CC alleles for these markers (Table 7). As an example, in Figure 4, we display the addition of both parental alleles in the SMC-58 somatic hybrid for the NTCP*7* SSR marker.

**Table 8:** Plant morphology of 9-month-old plants of the somatic hybrids SMC-58 and SMC-73 obtained by protoplast fusion between Carrizo citrange (CC) and *C. macrophylla* (CM).

**Genotype**	**Plant height (cm)**	**Leaf number**	**Internodal length (cm)**	**Leaf greenness (SPAD)**	**Leaf morphological index (l/w)**
CC	143.6a	41.9a	3.43a	73.82a	2.51a
CM	111.9b	36.8b	3.05b	60.93b	2.14b
SMC-58	59.1d	19.2d	3.08bc	72.78a	1.74c
SMC-73	81.5c	26.6c	3.14c	75.99a	1.79c

*Values are the mean of 12 plants (n = 12). Different letters within each column indicate significant differences for P ≤ 0.05 on multiple range Duncan's test*.

### Plant morphology

**Figure 5:**
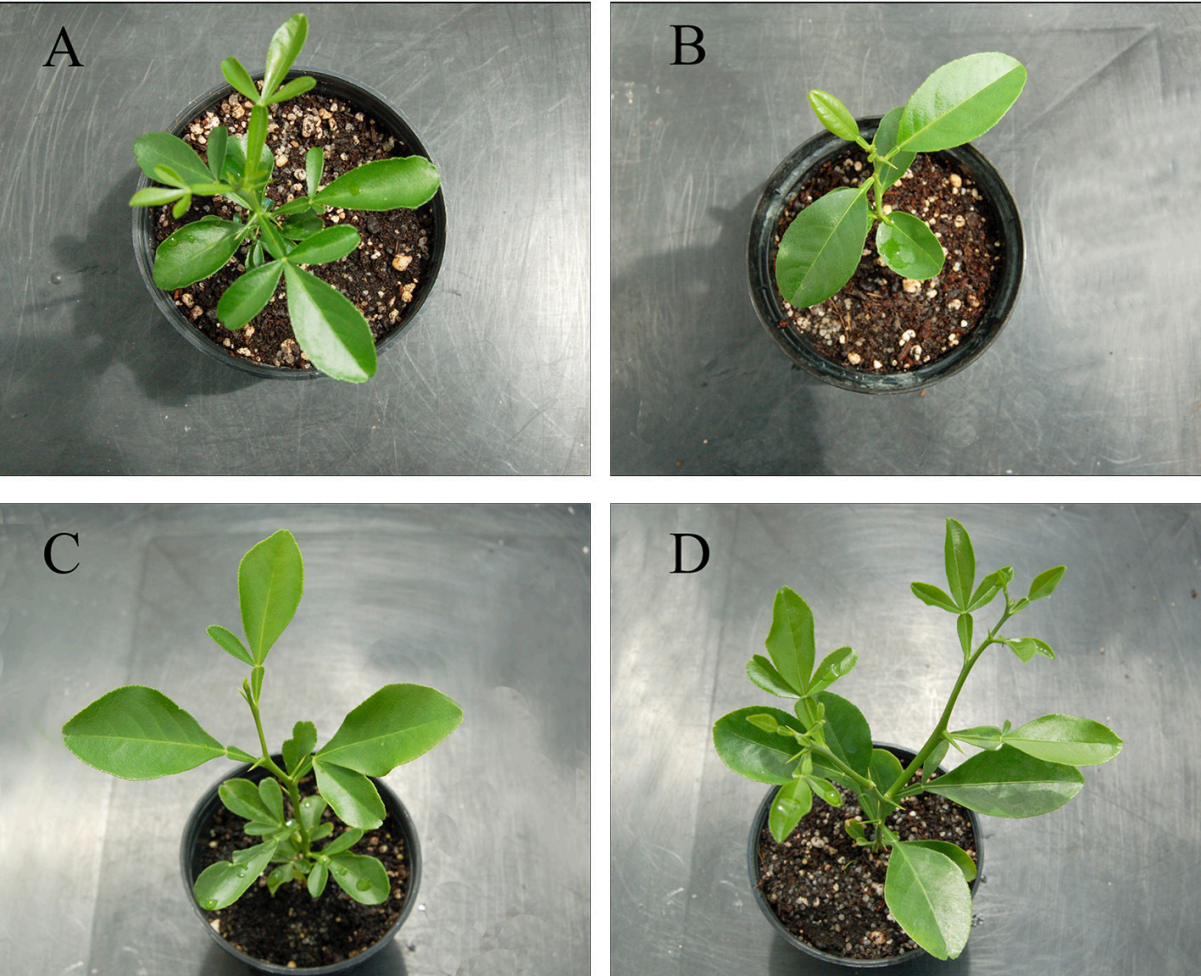
Plants of **(A)** Carrizo citrange (CC*)*, **(B)** *C. macrophylla* (CM) and somatic hybrids **(C)** SMC-58 and **(D)** SMC-73 cultivated for 5 months in greenhouse conditions.

Plant morphology was evaluated in 9-month-old plants of CC, CM, SMC-58, and SMC-73. Somatic hybrids had a slower growth than both the parents and were prone to lateral branching (Figures 5A–D). The height of both the somatic hybrids was shorter than CC or CM (Table 8). Differences in growth were also found between the hybrids as SMC-58 grew 20% less than SMC-73. Internodal length was longer in CC than in CM, whereas SMC-58 was similar to CM, and SMC-73 had an intermediate length between parents. The leaf morphological index obtained from the length/width ratio of the main leaflet was lower in somatic hybrids than in both the parents and was similar between SMC-58 and SMC-73. This indicates that the morphology of the measured leaves is round shaped, which is a character that is typical of tetraploid citrus plants (Barrett and Hutchison, 1978). Leaf greenness of somatic hybrids was similar between them and resembled CC, whereas CM had 12% lower leaf greenness than these genotypes. Somatic hybrid plants, SMC-58 and SMC-73, have a spiral phyllotaxis pattern, where leaves and straight thorns of intermediate length (16–40 mm) appear together. These characteristics are similar to those of both the parents. The leaves, showing brevipetiolate attachment to the lamina, are odd-pinnate, and the number of leaflets within the same plant varies between one, as seen with CM, and three, as seen with CC (Figures 5C and D). The somatic hybrid SMC-58 shows mainly one or two leaflets per leaf and trifoliate leaves are also present. The somatic hybrid SMC-73 shows mainly trifoliate leaves, even though simple and bifoliate leaves also appear. The leaf size is small (10–20 cm^2^) and heterogeneous. The petiole is shorter than the lamina and has narrow obdeltate wings with articulate junction to the lamina. The main leaflet has a length/width ratio between 1.5 and 1.8 and shape varies from elliptic, like in CC, to obovate, as in CM. The leaf margins are crenate, and the apex is obtuse in both somatic hybrids.

### Tolerance to iron deficiency

Leaf greenness decreased in all the genotypes under the chlorosis-inducing treatment (Ch). Carrizo citrange had a greater greenness decline than CM and the somatic hybrids showed intermediate values between parents (Table 9). In terms of growth, the shoot developed under the Ch treatment in somatic hybrids had similar leaf biomass than in CM, whereas these values were higher than in CC. In control conditions, SMC-73 had similar growth to that of CC and SMC-58 grew less than both parents. Iron concentration in the leaves developed under the Ch treatment was higher in CM than in CC and somatic hybrids had intermediate concentrations between them (Table 9).

**Table 9:** Leaf greenness (f:i), increase in shoot biomass (DW g) and iron concentration in shoot leaves (DW ppm) of Carrizo citrange (CC), *C. macrophylla* (CM) and the somatic hybrids SMC-58 and SMC-73 cultivated in greenhouse conditions for 10 weeks, either in control conditions (20 μM Fe-EDDHA) or in iron-deficient conditions (10% (v/v) CaCO_3_ 10 mM NaHCO_3_, 2 μM Fe-EDDHA).

**Treatment**	**Genotype**	**Leaf greenness (f/i)^X^**	**Increase in shoot biomass (DW g)**	**Iron concentration (ppm)**
Control	CC	0.84 a	1.36 b	48.3 a
	CM	1.11 b	1.76 c	46.7 a
	SMC-58	1.02 b	0.89 a	38.7 a
	SMC-73	0.89 ab	1.21 b	43.5 a
Iron-deficient	CC	0.30 a	0.42 a	16.5 a
	CM	0.70 c	0.56 b	33.1 b
	SMC-58	0.56 b	0.52 b	23.9 ab
	SMC-73	0.52 b	0.61 b	21.7 ab

X *SPAD final/initial, values below 1 indicate greenness decrease. Values are the mean of six plants (n = 6). Different letters in each column indicate significant differences for P ≤ 0.05 on multiple range Duncan's test*.

### Tolerance to salinity

**Figure 6:**
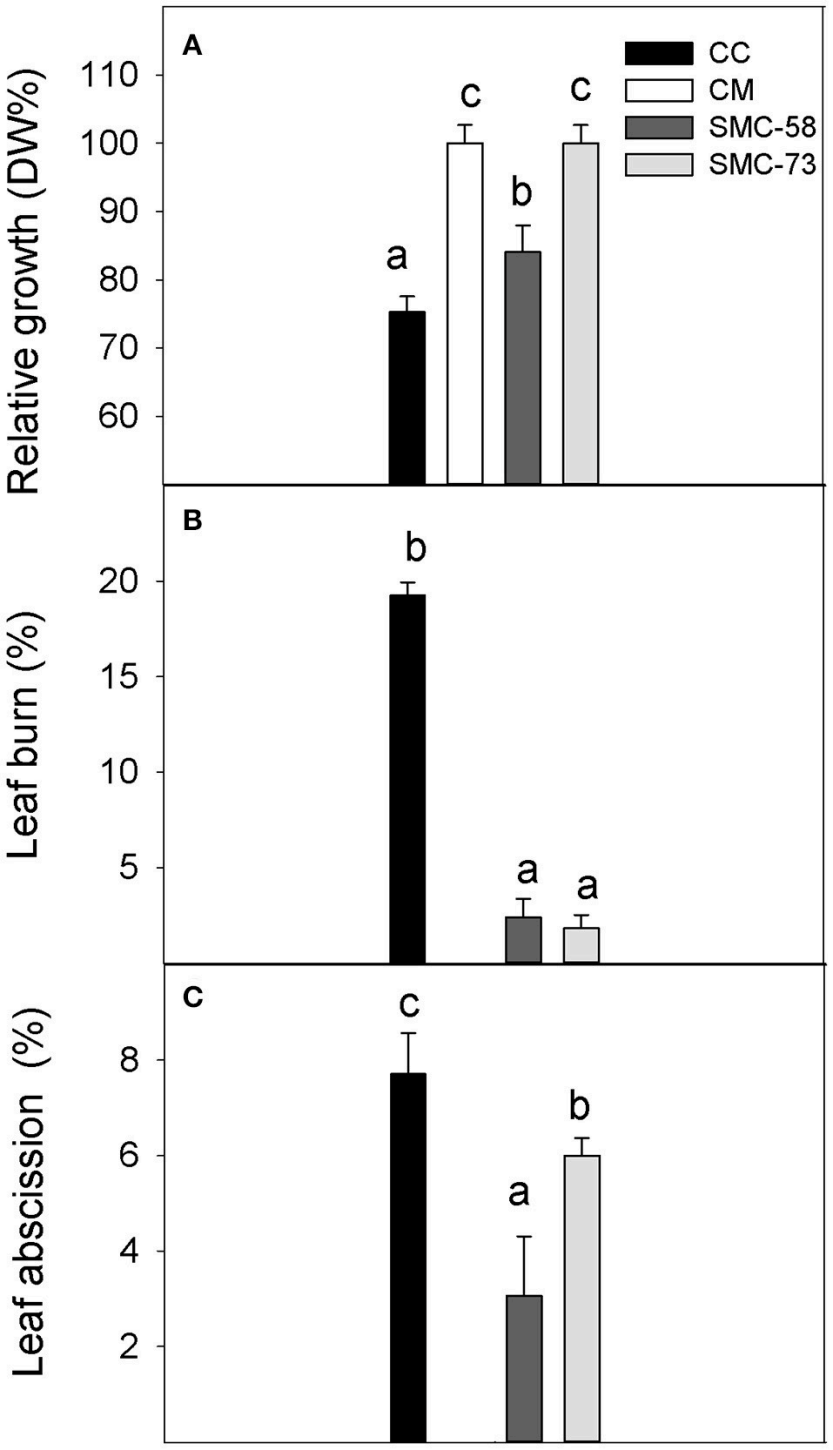
Influence of salinity on Carrizo citrange (CC), C. macrophylla (CM) and somatic hybrids SMC-58 and SMC-73 plants. **(A)** Relative growth (DW %), **(B)** Leaf burn damage (%) and **(C)** Leaf abscission. Plants were cultivated for 20 days under glasshouse conditions and saline treatment (40 mM NaCl). Values are the mean of six plants (*n* = 6) and standard error. Different letters indicate significant differences for *P* ≤ 0.05 on Duncan's multiple range test.

The differences in behavior between somatic hybrids and their parents under salinity (+S) were evaluated according to the growth rates, leaf symptoms, ion accumulation, and gas exchange parameters. Carrizo citrange plants subjected to salinity had 25% lower DW than control plants at the end of the experimental period (Figure 6A), indicating their sensitive behavior. In contrast, CM, that is salt-tolerant, showed similar growth in both, +S or Ct treatments. The SMC-73 hybrid had similar behavior to CM regarding growth, given that +S treatment did not affect this parameter. SMC-58 had 16% lower DW under the +S treatment than in Ct conditions, although this growth reduction was lower than in the sensitive CC. Leaf symptoms induced by salt toxicity were intense in CC plants that had 20% of their leaf area burned. Meanwhile, leaves of the tolerant CM were free of burns (Figure 6B) and somatic hybrids showed very mild leaf toxicity symptoms with only 2% (Figure 6C) of their leaf area affected by burns (Figure 6B). Leaf abscission was lower in SMC-73 or SMC-58 than in CC with 6, 3, and 7% of leaves affected, respectively. Nevertheless, the tolerant CM did not suffer this symptom.

Overall, Cl^−^ and Na^+^ molar concentrations in leaf tissue water were higher in the saline treatment (+S) than in the Ct treatment for all the genotypes (Table 10). The parent CM, which is tolerant to salinity, had the lower Cl^−^ and Na^+^ concentrations under the +S treatment, and were 2.2 and 1.4-fold, respectively, higher than in control plants. Carrizo citrange, which is considered salt-sensitive, raised Cl^−^ and Na^+^ leaf concentrations that were 4.1 and 2.6-fold higher in +S treatment than in Ct treatment, respectively. Somatic hybrid SMC-58 subjected to +S treatment had lower Cl^−^ concentration and similar Na^+^ concentration than CC. Specifically, Cl^−^ and Na^+^ leaf concentration in SMC-58 were 4.2 and 1.8-fold, respectively, higher in +S than in Ct plants. The SMC-73 plants subjected to +S treatment had leaf Cl^−^ concentrations similar to CC, whereas leaf Na^+^ concentration was higher than in CC plants. More precisely, leaf Cl^−^ and Na^+^ concentrations in SMC-73 increased by 3.9 and 2.3-fold, respectively, in salt-treated plants when compared to Ct plants. Therefore, the data show that both somatic hybrids had lower Cl^−^ exclusion capacity than the salt-tolerant parent CM. However, SMC-58 had greater exclusion capacity than the salt-sensitive parent CC, whereas SMC-73 had similar exclusion capacity to CC. Regarding Na^+^ exclusion, the behavior observed in SMC-58 was similar to CC, whereas SMC-73 plants accumulated less Na^+^ in their leaves, showing more tolerance than the sensitive parent CC. The concentration of K^+^ in plants subjected to salinity was not different from Ct plants in the tolerant CM. Leaf concentration of K^+^ decreased by 21%, 20%, and 33%, respectively, in CC, SMC-58, and SMC-73. Salt-treated CM plants did not differ from Ct plants in their *A*_CO2_ and *E* rates (Table 11). The salt-sensitive parent CC had reduced *A*_CO2_ rates by 39% and *E* rates by 18% when compared to Ct plants. Somatic hybrids SMC-58 and SMC-73 subjected to salinity reduced *E* rates by 33% and 43%, respectively, when compared to Ct plants. Similarly, these salt-treated plants reduced *A*_CO2_ by 32% and 53%, respectively. The data show that gas exchange parameters were more affected by salinity in SMC-73 than in SMC-58. Therefore, the former genotype was similar to CC, whereas the latter had a behavior more similar to CM. In summary, results show that somatic hybrids have an intermediate behavior between the tolerant rootstock CM and the sensitive CC. However, the differences found between SMC-58 and SMC-73 indicate that SMC-58 is better adapted to salinity than SMC-73 because the response was globally more similar to the tolerant parent CM.

**Table 10:** Leaf Cl^−^, Na^+^ and K^+^ concentration (mM in tissue water) in Carrizo citrange (CC), *C. macrophylla* (CM) and SMC-58 and SMC-73 somatic hybrids.

**Treatment**	**Ion**	**CC**	**CM**	**SMC-58**	**SMC-73**
Control	Cl^−^	56.3a	36.5a	43.5a	53.4a
	Na^+^	140.0b	85.7a	217.9c	202.0c
	K^+^	571.1a	607.0b	556.2a	609.0b
Saline	Cl^−^	233.2c	80.9a	183.0b	205.4c
	Na^+^	367.8b	117.5a	393.7b	466.0c
	K^+^	462.0a	621.2b	446.0a	468.4a

*Values are the mean of 3 plants (n = 3). Different letters in each line indicate significant differences for P ≤ 0.05 at Duncan's multiple range test. Plants were cultivated in a greenhouse for 20 days, either in saline (40 mM NaCl) or control conditions*.

**Table 11:** Transpiration (*E*, mmol H_2_O·m^−2^·s^−1^) and net assimilation (*A*_CO2_, μmol CO_2_·m^−2^·s^−1^) rates in Carrizo citrange (CC), *C. macrophylla* (CM) and SMC-58 and SMC-73 somatic hybrids.

**Treatment**	**Parameter**	**CC**	**CM**	**SMC-58**	**SMC-73**
Control	*E*	0.76 c	1.37 a	1.32 a	1.16 b
	*A*_CO2_	6.23 b	9.99 a	9.21 a	8.74 a
Saline	*E*	0.62 c	1.32 a	0.89 b	0.66 c
	*A*_CO2_	3.82 c	10.1 a	6.23 b	4.14 c

*Values are the mean of six plants (n = 6). Different letters in each line indicate significant differences for P ≤ 0.05 at Duncan's multiple range test. Plants were cultivated in a greenhouse for 20 days, either in saline (40 mM NaCl) or control conditions*.

### Tolerance to CTV

**Figure 7:**
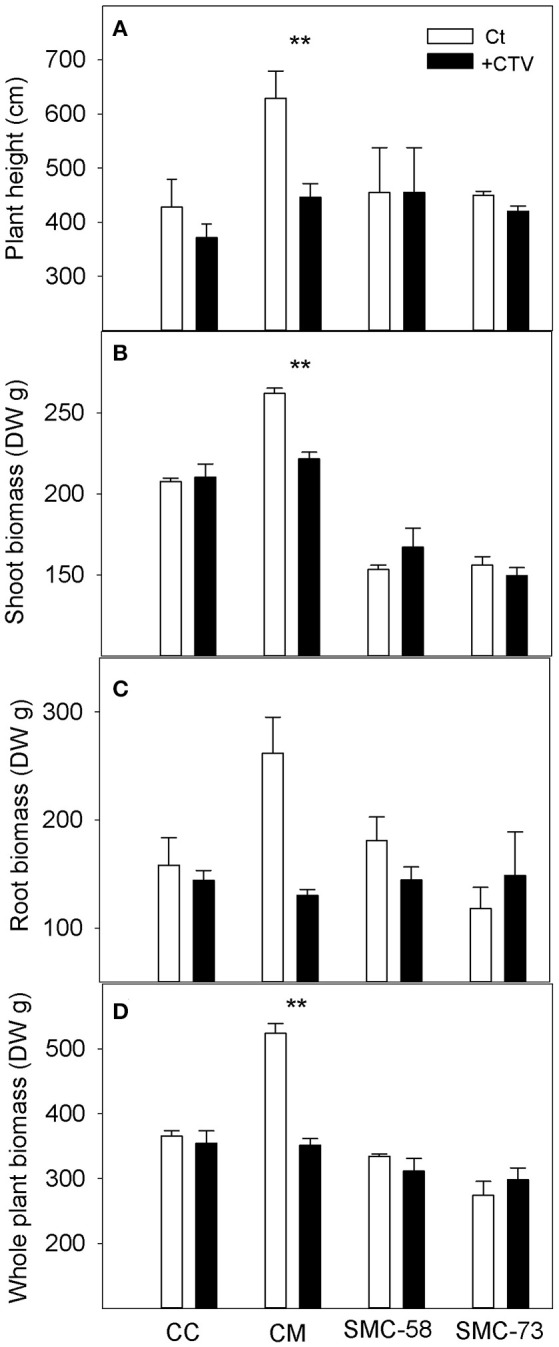
Influence of *Citrus tristeza virus* (CTV) on Carrizo citrange (CC)*, C. macrophylla* (CM) and somatic hybrids SMC-58 and SMC-73 plants. **(A)** Plant height (cm), **(B)** Aerial parts plant biomass (DW g), **(C)** Root biomass, **(D)** Whole plant biomass. Plants were either inoculated with the T388 strain of CTV (+CTV) or treated as non-inoculated controls (Ct) and cultivated for 12 months under glasshouse conditions. Values are the mean between 3 and 6 plants and standard error. **Significant differences for *P* ≤ 0.01 on Duncan's multiple range test.

**Figure 8:**
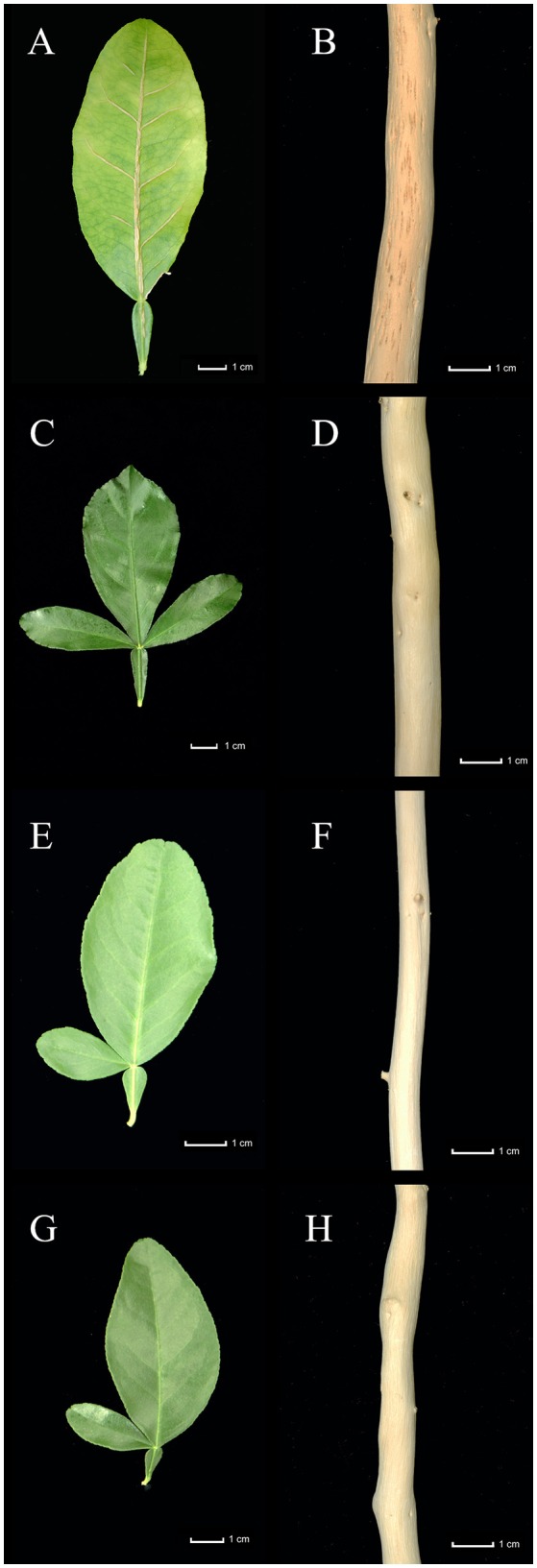
Symptoms on plants inoculated with the T388 strain of *Citrus tristeza virus*: **(A)** Yellowing and vein corking in leaf of *C. macrophylla* (CM). **(B)** Stem pitting in CM. **(C,D)** Symptomless leaf and stem of Carrizo citrange. **(E,F)** Symptomless leaf and stem of somatic hybrid SMC-58. **(G,H)** Symptomless leaf and stem of somatic hybrid SMC-73.

Plants inoculated with T388 CTV strain were evaluated for growth and symptoms. CM showed reduced growth as evident by the shorter height of the plants (Figure 7A) and the lower aerial and root biomass (Figures 7B and C). Overall, plant biomass decreased by 35% (Figure 7D) in CM. These plants also showed yellow leaves with vein corking (Figure 8A) and stem pitting (Figure 8B). Meanwhile, CC plants and the somatic hybrids SMC-58 and SMC-73 were not different from control plants in their growth (Figure 7D), and neither showed the disease symptoms (Figures 8C–H).

## Discussion

### Genetic characterization of the somatic hybrids

Two allotetraploid somatic hybrids were previously obtained from protoplasts isolated from callus of CM and from leaves of CC (Pensabene-Bellavia et al., 2015). These somatic hybrids, their parents, and the embryogenic callus of CM were analyzed to verify their origin and genetic structure of the hybrids. Fifty-six nuclear molecular markers distributed uniformly on the 9 LGs of the clementine genetic map (Ollitrault et al., 2012a) and 5 cytoplasmic markers that were polymorphic between the parents were used for the analysis. Nuclear molecular markers confirmed that (i) the embryogenic callus of CM did not show differences compared to leaves of CM for all these markers and (ii) the somatic hybrids added the parental genomes although some parental alleles were lost. Specifically, 11 lost alleles have been identified on different LGs (2, 3, 6, and 7) although most of them are located on LG 6 (8 of the 11 alleles). Differences in 5 markers have also been found between the somatic hybrids. Most of the lost alleles observed in the somatic hybrids have their origin in the embryogenic parent CM (7 of them) even though 4 of them correspond to the leaf parent CC. For some markers, it has been possible to identify the parental origin of the lost allele. Most of the lost alleles come from *C. medica*, whereas others come from *C. micrantha, C. sinensis*, and *P. trifoliata*. These results suggest that the loss of parental alleles occurred during the somatic hybridization process and the differences observed between hybrids seem to be limited to sub-chromosomal level because the flow cytometry analysis did not show differences in the number of chromosomes (2n = 4x = 36) between somatic hybrids or when compared to the tetraploid control. Furthermore, genetic analysis has been performed using leaf samples of the somatic hybrids taken from different branches and the same differences between somatic hybrids and parents were found. However, no differences were identified between analyses of different DNA preparations, discarding the presence of chimeras. Most of the absent alleles that have been identified are located on the same LG and consist of deleted fragments (SSR allele absence) and punctual variations in a small number of nucleotides (non-observed SNP alleles). This indicates that there is chromosome instability in this complex intergeneric combination given that the genomes of the four citrus ancestral species and related genera are present in the somatic hybrids. Previous studies performed in citrus (Xu et al., 2014) and other species (Sundberg and Grimelius, 1991; Sun et al., 2014; Smyda-Dajmund et al., 2016) state that chromosome losses, genomic deletions, and epigenetic alterations are more frequent in somatic hybrids between parents that have a distant genetic relationship than in those from closely related parents. In SMC-58 and SMC-73, most lost alleles do not come from the species that are genetically more distant, *C. micrantha* and *P. trifoliata*. This finding suggests that there is no bias against the most dissimilar genomes when somatic hybridization is performed. Therefore, the identified losses might be either random or caused by some other effect. It has also been reported that genomic losses in citrus somatic hybrids are parent-biased toward the callus parent (Xu et al., 2014), which might explain that most of the alleles lost in SMC-58 and SMC-73 come from CM. Overall, the wide diversity of the genomes combined in SMC-58 and SMC-73 and the different origin of the parental protoplasts used to perform the fusions might explain the uneven genomic losses that we observed. Nevertheless, further studies would be required to verify these hypotheses.

Differences between SMC-58 and SMC-73 have also been found in the cytoplasmic genome. Both hybrids have the CM mitochondrial genome. However, SMC-73 has the CC chloroplastic genome, whereas chloroplastic genome recombination was detected in SMC-58. Citrus somatic hybrids predominantly inherit the mitochondrial genome from the embryogenic parent (Kobayashi et al., 1991; Saito et al., 1993; Yamamoto and Kobayashi, 1995; Moriguchi et al., 1997; Moreira et al., 2000; Cabasson et al., 2001; Ollitrault et al., 2001; Guo et al., 2002; Xiao et al., 2014) even though there are some reports of mitochondrial recombination events (Vardi et al., 1987; Moriguchi et al., 1997; Cheng et al., 2003; Dambier et al., 2011). Recently, Cai et al. (2017) have demonstrated that mitochondrial genome of protoplasts isolated from embryogenic callus is essential for plant regeneration after protoplast fusion experiments. However, chloroplastic genome is randomly inherited from one of the parents or shows recombination (Grosser et al., 2000; Dambier et al., 2011; Aleza et al., 2016). It has also been proven that mitochondrial and chloroplastic genomes are involved in differences in aroma and organoleptic fruit properties (Fanciullino et al., 2005; Satpute et al., 2015), disease resistance (Tusa et al., 2000; Omar et al., 2017), floral developmental disturbances, and male sterility (Guo et al., 2004; Zheng et al., 2012). Nevertheless, there is no information available about how these new combinations and rearrangements occurring on the cytoplasmic genome affect the agronomical behavior of citrus rootstocks. Most publications on citrus somatic hybridization report the symmetric addition of nuclear parental genomes (Ollitrault et al., 2010a) although subchromosomal variations have been detected in citrus somatic hybrids (Olivares-Fuster, 1998; Froelicher, 1999; Guo et al., 2007; Xu et al., 2014). However, studies describing them are scarce. Different hypotheses have been suggested for these kind of changes such as extended periods of *in vitro* culture (Oberwalder et al., 1998; Guo and Deng, 1999), genetic divergence between parents, and increased ploidy level (Sundberg and Grimelius, 1991; Miranda et al., 1997). Genetic analysis of somatic hybrids has been usually performed with a small number of molecular markers, enough to confirm their hybrid origin but not sufficient to identify these variations (Oberwalder et al., 1998; Guo and Deng, 1999). In potato (*Solanum* spp) somatic hybrids, similar variations have also been recently described using DArT markers (Diversity Array Technology) (Smyda-Dajmund et al., 2016). More than 5,000 markers distributed across the potato genome were analyzed in the somatic hybrids and 2,000 were found to be polymorphic between parents. Among them, between 13.9% and 29.6% of alleles were found to be lost in the somatic hybrids. The identification of genomic changes in somatic hybrids justifies the need for performing a detailed genetic analysis of the plants obtained by somatic hybridization to gather information on their genetic structure. This information is key to optimize and interpret the data on physiological behavior of the somatic hybrids to use them as rootstocks.

### Performance of SMC-58 and SMC-73 somatic hybrids as potential citrus rootstocks

Several rootstock breeding programs based on somatic hybridization are currently being carried out across the world. In Florida, a large number of somatic hybrids have been obtained, which stand out for their good adaptation to the local soil, inducing good fruit quality, and high yields (Grosser et al., 2015). Breeding programs focused on somatic hybridization have also been carried out in the Mediterranean basin (Dambier et al., 2011), as well as in China (Guo et al., 2002, 2007), Brazil (Mendes-da-Gloria et al., 2000; Mourao et al., 2008), and Mexico (Medina-Urrutia et al., 2004). These data reveal that somatic hybridization is an efficient approach to produce new citrus rootstock candidates. The morphology of somatic hybrids SMC-58 and SMC-73 shows some intermediate characters between the parents. This type of inheritance has also been described in somatic hybrids between *Citrus* and related genera such as *Citropsis, Severinia*, and *Microcitrus* (Smith et al., 2013) and also between different *Citrus* species (Olivares-Fuster, 1998). The growth of somatic hybrids when compared with their parents is slower as it has also been described in several citrus allotetraploid somatic hybrids (Grosser et al, 1998; Grosser et al., 2012). This character is related to the increase in ploidy level (Lee, 1988, 1990). Citrus tetraploid hybrids have been used to increase the tree density in orchards to maximize the management efficiency. Furthermore, tetraploid rootstocks do not reduce the yield efficiency of the scion (Ruiz et al., 2016a) and produce fruits with excellent organoleptic qualities (Grosser et al., 2015). Therefore, the profitability of citrus plantations can be increased using tetraploid citrus rootstocks (Grosser et al., 1995, 2015; Grosser and Chandler, 2003).

We have evaluated the tolerance/susceptibility of CM + CC somatic hybrids to the severe T388 CTV strain. This strain causes different symptoms in susceptible citrus genotypes. The symptoms include seedling yellows, vein corking, or stem pitting when used either as varieties or rootstocks and the quick-decline of trees grafted onto SO (Moreno et al., 2008; Lee and Keremane, 2013). CM was found very sensitive to T388, whereas CC and the two CM + CC somatic hybrids were found to be tolerant. In Spain and in other Mediterranean countries, severe strains of CTV have been identified even though the incidence is low (Moreno et al., 2008). However, *Toxoptera citricida* (Kirkaldy), which is a very efficient vector of severe CTV strains, is already present in northern Portugal and north western Spain. The probable introduction of this aphid into the citrus producing areas would predictably cause a dispersion of severe CTV strains that would affect the trees grafted onto CM (Ilharco et al., 2005). Therefore, it is very important to have alternatives to this rootstock that can be used in alkaline and saline soils, where CC is not a good choice. In other studies, the quick decline was evaluated in somatic hybrids obtained from SO and several tolerant species, but global conclusions could not be reached. While somatic hybrids between SO and Rangpur lime (*C. limonia* Osb.) or Rough lemon (*C. jambhiri* Lush) were tolerant, hybrids obtained from SO and trifoliate orange (*P. trifoliata*) or Cleopatra mandarin were susceptible to this disease (Grosser et al., 1996). The inheritance of some traits such as CTV tolerance in the somatic hybrids is clearly coupled with the dominance or codominance of the trait in relation to the parental combinations (Bassene et al., 2009; Gmitter et al., 2012).

The performance of the somatic hybrids in the presence of soil carbonates, which are abundant in the Mediterranean citrus producing areas, is similar to the tolerant CM and much better than CC. The CM + CC hybrids are also more tolerant to salinity than CC. Enhanced tolerance to these stresses as well as to drought and boron excess has also been described in citrus rootstocks with increased ploidy (Saleh et al., 2008; Grosser et al., 2012; Allario et al., 2013; Tan et al., 2015; Ruiz et al., 2016a,b,c). These somatic hybrids have already started to yield fruits even though fruits are still sporadic and scarce. A large number of apomictic seeds per fruit were found. This characteristic is very important for citrus rootstocks as their clonal propagation and cultivation in nurseries are made easy. Field experiments have already been initiated in collaboration with Agromillora Research S.L. and will allow, within a few years, to confirm the data obtained in the greenhouse experiments and to collect additional information about fruit quality and yield induced by the grafted variety. All this information will be analyzed to determine if any of the studied somatic hybrids can be used commercially, which would be a great advantage for the Mediterranean citriculture.

### The importance of performing in-depth molecular and physiological characterization of somatic hybrids

The main goal of citrus rootstock improvement based on somatic hybridization by protoplast fusion is to recover allotetraploid somatic hybrids between parents displaying complementary characteristics as seen in our study. Previous studies on somatic hybridization variability carried out in the past decades reveal that characters expressed by the hybrids can be non-additive. The hybrid phenotype can differ from the addition of parental effects given that allopolyploidization triggers gene expression changes and modifies epigenetics altering the phenotype (Bassene et al., 2009, 2010; Dambier et al., 2011; Xu et al., 2014). Some studies discuss to what extent these changes are caused by *de novo* interactions established between genomes coming from different species (Hegarty et al., 2009) or to the ploidy gain (Dambier et al., 2011; Tan et al., 2017). Allopolyploidization, generated either by sexual or somatic hybridization, involves the coexistence of parental genomes in a single nucleus. Additionally, in the case of allotetraploid somatic hybrids, changes also take place in cytoplasmic genome composition. The new genomic configuration is associated with diverse reorganizations and modifications affecting the structure and regulation of the new somatic hybrid genome (Comai et al., 2000; Ozkan et al., 2001; Wang et al., 2006a; Soltis and Soltis, 2009; Flagel and Wendel, 2010). This event, coined as genomic *shock* (Song et al., 1995), has a dynamic and stochastic nature and is composed of diverse processes such as fragment elimination or exchange at sub-chromosomic or chromosomic level, modifications in the methylation pattern, gene repression/expression changes, and activation of transposable elements (Chen, 2007; Xu et al., 2014) among others. These changes modify the gene expression either by altering the sequence or by epigenetic regulation (Comai, 2005). In addition, these changes may confer genome plasticity to improve the adaptation of the hybrids to the environment (Chen, 2007). The neoregulation of parental genomes in allopolyploid plants would greatly explain the obtention of genotypes and phenotypes that were absent in the diploid pool (Osborn et al., 2003) and the non-additive inheritance (He et al., 2003; Albertin et al., 2006; Hegarty et al., 2006; Wang et al., 2006a,b; Chen, 2007, 2010; Flagel et al., 2008; Flagel and Wendel, 2010). The study of genome expression in neopolyploids has recently gained importance, as it has been proposed as a useful approach to understand how genomes work and evolve (Gmitter et al., 2012; Gianinetti, 2013).

Somatic hybridization is more efficient than sexual hybridization as a method for citrus breeding when parents display a complex reproductive biology such as apomixis and high heterozygosity, as in the case of most of the rootstocks used. Nevertheless, it is still necessary to regenerate an adequate number of plants from each fusion to perform further screenings that verify their characteristics and agronomic behavior. This is essential to properly assess their usefulness in breeding programs, yet, the molecular basis of the traits that shape the rootstock agronomical behavior is still unknown. The new genetic (Ollitrault et al., 2012a) and genomic (Wu et al., 2014) tools available nowadays along with the affordable sequencing technologies are paving the way for the availability of numerous molecular markers and genetic information. This knowledge will contribute to the understanding of the molecular processes behind these traits and shorten the time required to perform additional evaluations. It is also essential to have rapid screening methods for early evaluations in greenhouse conditions. This will maximize the efficiency of breeding programs in terms of time, resources, and labor costs. Only those traits that are strictly necessary should be considered for long-term field evaluations.

## Conclusion

Somatic hybrids SMC-58 and SMC-73 are promising citrus rootstocks for areas with the presence of CTV and calcareous and saline soils. They have punctual sub-chromosomic losses and show differences in morphology and physiological behavior, both between them and when compared with their parents. This is an evidence of genomic alterations that affect each hybridization event individually and are somehow independent from parental combinations. These identified genetic variations, along with the possible neoregulation events, the new cytoplasmic combinations, and the ploidy gain, might be the underlying phenotypic differences found between the hybrids and the phenotypic deviation from parental additive inheritance. Further investigation on somatic hybrids can add great value to citrus breeding programs as it can reveal information crucial to understand the principles operating in citrus genome expression, regulation, and evolution.

## Author contributions

LN, EP-M, PO, and RM conceived the study and were in charge of the direction and planning. MR, AQ, GP-B, EP-M, LN, and RM contributed in the experiment design. MR, AQ, GP-B, and AG-L performed the experiments. MR, AG-L, and PA analyzed the data. MR and PA took the lead in interpreting the results and writing the manuscript with input and review from LN, EP-M, and PO.

### Conflict of interest statement

The authors declare that the research was conducted in the absence of any commercial or financial relationships that could be construed as a potential conflict of interest.
